# The impact of COVID-19 on male reproductive health: a Systematic Review

**DOI:** 10.5935/1518-0557.20240028

**Published:** 2024

**Authors:** Beatriz Matos Anastácio, Paula Bruno Monteiro, Melissa Figueiredo Capelo

**Affiliations:** 1 Christus University Center (UNICHRISTUS), Biomedicine Department. Fortaleza, Ceará, Brazil

**Keywords:** infertility, pandemic, assisted reproduction, COVID-19

## Abstract

**Objective:**

This systematic literature review aims to assess the impact of COVID-19 on male fertility

**Data Sources:**

The study draws upon data extracted from PubMed, SciELO, and LILACS databases.

**Study Selection:**

The review incorporates cross-sectional studies, cohort studies, and clinical trials, encompassing investigations related to the subject matter. The studies included were published between June 2020 and March 2023, and encompassed content in English, Portuguese, and Spanish. Exclusion criteria encompassed review articles, case reports, abstracts, studies involving animal models, duplicate articles, and letters to the editor.

**Data Collection:**

Data extracted included the author’s name and publication year, the number of patients studied, patient age, the presence of COVID-19 in semen, observed hormonal changes, and alterations in seminal quality.

**Conclusions:**

While hormonal changes and a decline in seminal quality were observed in COVID-19 patients, the virus itself was not detected in semen in the analyzed articles, which contradicts certain findings in the existing literature. It is essential to note that methodologies in the studies were diverse, and, due to the novelty of this infection, it is premature to definitively ascertain its long-term effects on male fertility or whether fertility can recover after a period of convalescence. This underscores the necessity for further research, utilizing more robust methodologies such as cohort studies.

## INTRODUCTION

Infertility is a significant global health concern, with estimates from the World Health Organization (WHO) indicating that around one in six individuals of reproductive age grapple with infertility during their lifetime. In the context of male reproductive health, infertility primarily arises from issues related to semen ejection, the presence or low concentrations of sperm, deviations in sperm morphology, or reduced sperm motility (WHO, 2023).

Beyond these common factors, male infertility can also be influenced by a variety of other contributors, including environmental influences, mechanical factors, genetic disorders, failures in spermatogenesis, and infections. Notably, the emergence of the severe acute respiratory syndrome coronavirus 2 (SARS-CoV-2) and the ensuing global pandemic, Coronavirus Disease 2019 (COVID-19), spanning from 2019 to 2023, brought forth new considerations in the realm of male reproductive health (Lima & Lourenço, 2016).

The COVID-19 pandemic, which emanates from the SARS-CoV-2 virus, has had a profound impact on public health, resulting in millions of confirmed cases and fatalities across the world (Safiabadi Tali *et al.*, 2021). Initially regarded as primarily a respiratory virus, SARS-CoV-2 demonstrated a strong affinity for the angiotensin-converting enzyme 2 (ACE2) receptor, which is highly expressed in pulmonary tissues and serves as the portal for viral entry into human cells (Rodrigues & Dantas, 2022).

Nevertheless, emerging research has expanded our understanding of the distribution of ACE2 receptors, revealing their presence not only in the lungs but also in other vital organs, including the heart, liver, and notably, the male reproductive system. Studies have underscored the elevated expression of ACE2 in the testes, particularly in spermatogonia, Leydig cells, and Sertoli cells, indicating a heightened susceptibility of the testes to SARS-CoV-2 infection, which could have implications for male fertility (Fernandes *et al*., 2022; Rodrigues & Dantas, 2022). Consequently, this study seeks to undertake a comprehensive literature review to evaluate the impact of COVID-19 on male fertility.

## MATERIALS AND METHODS

### Study design

This study constitutes a systematic literature review, conducted in accordance with the guidelines outlined in the Preferred Reporting Items for Systematic Reviews and Meta-Analyses (PRISMA) framework.

### Search strategy

In order to initiate our search, we initially identified relevant Medical Subject Headings (MeSH) descriptors. Subsequently, these descriptors were integrated with Boolean operators to create three distinct search strategies: (1) “spermatogenesis AND male infertility AND pandemic COVID-19”; (2) “spermatogenesis AND COVID-19 pandemic”; and (3) “male infertility AND pandemic COVID-19”. These search strategies were executed across three comprehensive databases: PubMed, SciELO, and LILACS.

### Study eligibility criteria

To select relevant studies for our review, we implemented the following eligibility criteria: clinical trials, cohort studies, and cross-sectional studies that addressed the specific theme proposed by the authors. The selected studies included in this review were limited to those published within the timeframe of June 2020 to March 2023, and encompassed content published in English, Portuguese, and Spanish.

### Inclusion criteria

- Cohort studies
- Cross-sectional studies
- Clinical trials

### Exclusion criteria

- Review articles
- Letters to the editor
- Abstracts
- Studies conducted on animals
- Duplicated articles

### Screening and selection process

Following the completion of our searches, initial screening was conducted based on the titles and abstracts of retrieved articles. Any studies failing to meet the aforementioned criteria were excluded. When an article met the defined criteria for inclusion, it underwent assessment by two independent authors (BMA and MFC). In cases where both authors reached a consensus regarding the clinical relevance of a study, the corresponding data were subsequently tabulated. In instances of dissent, a third author, a specialist in the relevant field (PMB), was consulted for a final resolution.

### Data extraction

From the articles that passed through the screening process, the following data were systematically extracted:

- Author’s name
- Number of patients
- Patient age
- Timing of semen sample collection and analysis
- Presence of COVID-19 in semen
- Hormonal alterations
- Changes in seminal quality

## RESULTS

### Characteristics of the included studies

**Figure 1:**
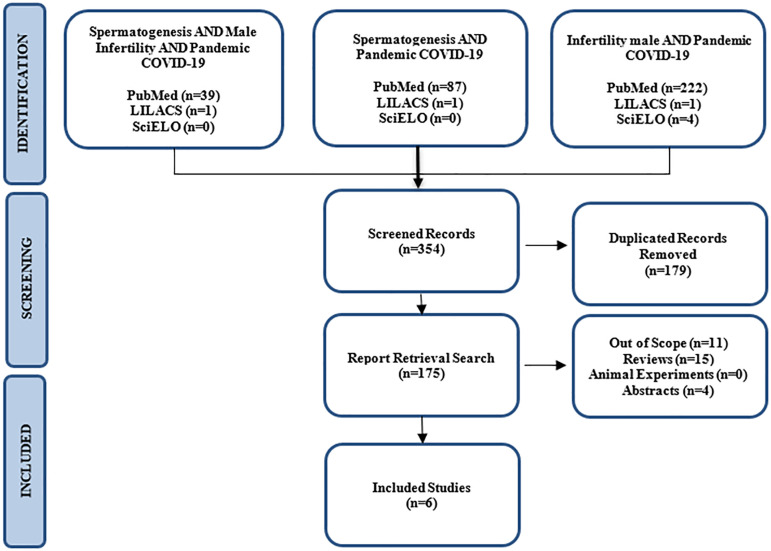
Methodological flowchart.

Our data search yielded a total of 160 records across PubMed (n=159), SciELO (n=0), and LILACS (n=1). After careful screening and selection, six articles met the criteria for inclusion in this review, as illustrated in Figure 1. The age range of the patients in these selected studies varied from 26 to 42 years. The extracted data have been systematically organized in Table 1.

**Table 1:** Included studies characteristics.

Author(s), Year	Number of Patients	Semen Collection and Analysis	COVID-19 in Semen	Seminal Quality Changes	Hormonal Changes
Best *et al*., 2021	30 men	Median time of 3 months after treatment and recovery	Not detected in any semen samples tested	↓ Concentration and total number of spermatozoa; pH and normal volume	-
Guo *et al*., 2021	1st Sample: 41 men 2nd Sample: 22 men	Median time of 76.0 days after the appearance of symptoms and 56.0 days after hospital discharge	-	27 men (65.9%): abnormal value for at least one semen parameter. 1st Sample ↑ Asthenozoospermia; ↓ Progressively motile or motile spermatozoa; Volume, vitality, abnormal morphology 2nd Sample: ↑ Total sperm count, concentration and motile sperm count compared to the first sample	Regular T, LH, FSH and AMH ↑ Prolactin ↓ Progesterone ↓ Inhibin B in 13 of the 38 patients but without statistical significance
Gul *et al*., 2021	29 men	The median duration between testing positive for SAR-CoV-2 and semen collection was 37 days	-	Volume, Full motility, Progressive motility, Concentration, and total number of spermatozoa normal	Regular T, LH, FSH and Prolactin.
Holtmann *et al*., 2020	34 men	18 semen samples were obtained 8-54 days after absence of symptoms, 14 from control subjects and 2 from patients with active COVID-19 infection. Not detected in recovered men or men with acute COVID-19 infection	Not detected in recovered men or men with acute COVID-19 infection.	↓ Volume; ↓ Total number and concentration of sperm ↓ Progressive and complete motility ↑ Immobile spermatozoa	-
Koç & Keseroğlu, 2021	21 men	None of the patients were in the active phase of COVID-19 at the time of semen collection	-	↓ Volume; ↓ Total motility; ↓ Progressive motility ↓ Normal morphology ↑ Immobile spermatozoa	↓ Testosterone FSH and Regular LH
Pan *et al*., 2020	34 men	Median time of 31 days since COVID-19 diagnosis	Not detected in recovered patients	-	-

T: Testosterone; FSH: Follicle Stimulating Hormone; LH: Luteinizing Hormone; and AMH: Anti-Mullerian Hormone.

### Presence of COVID-19 in semen

Within the scope of this review, we identified that only three studies examined the presence of viral RNA in the semen of men who had contracted SARS-CoV-2. Remarkably, across these three studies, the median duration between a positive SARS-CoV-2 test and semen collection was strikingly similar, with intervals of 31 days, 37 days, and 32 days. Notably, the virus was not detected in the semen of any of the men who had recovered from COVID-19 (Best *et al.*, 2021; Holtmann *et al.*, 2020; Pan *et al.*, 2020).

### Changes in seminal quality

Seven studies were scrutinized in our quest to assess whether there were alterations in seminal quality among men who experienced COVID-19 during the pandemic period. Among these studies, a single investigation reported no significant variations in spermogram results (Gul *et al.*, 2021). However, the remaining studies revealed abnormal values for at least one semen parameter, emphasizing a potential impact on seminal quality (Best *et al*., 2021; Guo *et al*., 2021; Holtmann *et al*., 2020; Koç & Keseroğlu, 2021).

### Hormonal changes in men with SARS-CoV-2 infection

Our analysis disclosed that only three studies provided insights into potential changes in serum levels of sex hormones among men diagnosed with COVID-19. Notably, one of these studies reported no hormonal alterations (Gul *et al.*, 2021). In contrast, the remaining studies revealed varied hormonal changes, with one reporting a low testosterone level (Guo *et al.*, 2021) and another noting an increased prolactin level and decreased progesterone (Koç & Keseroğlu, 2021).

## DISCUSSION

The infection caused by COVID-19 is known to trigger an excessive and unregulated release of early response pro-inflammatory cytokines while also elevating oxidative stress through an augmented production of reactive oxygen species (ROS). The interplay between elevated ROS and pro-inflammatory cytokines, as discerned from an analysis of five articles examined in this study, appears to exhibit a potential negative correlation with seminal quality parameters.

Moreover, observations within the male patient cohort convalescing from COVID-19 reveal a distinctive trajectory. Initial impairment in seminal parameters at the onset of infection progressively trends toward normal values over time, as observed by Hajizadeh Maleki & Tartibian (2021).

However, within the extant literature, two studies, methodologically inclined toward assessing the broader repercussions of the pandemic as opposed to specifically investigating the impact of COVID-19 infection, were identified. Notably, neither the study by Sarier *et al.* (2022) nor the study by Kumar *et al.* (2023) diagnosed any patients with COVID-19, and both revealed no significant disparities in spermogram results. Curiously, Kumar *et al.* (2023) further demonstrated that in their pandemic group, the median sperm concentration, total sperm count, and total motility surpassed the pre-pandemic cohort.

Conversely, the preponderance of studies encompassed in this review concentrated on patients confirmed to have contracted COVID-19 and, in contrast, yielded conflicting outcomes. Two studies, led by Best *et al.* (2021) and Guo *et al.* (2021), disclosed significantly reduced sperm concentration and total sperm count in SARS-CoV-2 positive individuals when compared to their SARS-CoV-2 negative counterparts.

Another investigation underscored a reduction in semen volume, total motility percentage, progressive motility percentage, and normal sperm morphology in COVID-19-afflicted patients when contrasted with non-infected individuals (Koç & Keseroğlu, 2021). In the analysis by Holtmann *et al.* (2020), recovered COVID-19 patients were categorized as having experienced mild or moderate infections. Results illuminated that patients with moderate infections exhibited lower sperm volume, sperm concentration, total sperm count per ejaculate, total progressive motility, and total complete motility, alongside a higher prevalence of immobile sperm, when juxtaposed against their mild infection counterparts and the control group. These findings suggest a direct relationship between COVID-19’s effects on seminal quality parameters and the severity of the disease.

Furthermore, as elucidated in existing literature, testosterone, the principal androgen regulating spermatogenesis in the testes, is predominantly synthesized by Leydig cells in response to luteinizing hormone (LH) stimulation (Smith & Walker, 2014). This prompts the assertion that SARS-CoV-2 may exert an impact on Leydig cells via ACE2 receptors, potentially leading to diminished testosterone levels (T) and consequent repercussions on spermatogenesis, ultimately culminating in alterations in seminal parameters.

In alignment with the literature, the study conducted by Koç & Keseroğlu (2021) substantiated a significant decline in T levels following COVID-19 diagnosis. Despite limitations in participant numbers assessed for hormone levels, these findings are invaluable for presenting group analysis results contingent on a hormone, such as T, known for its considerable population-level variations. On the other hand, Gul *et al.* (2021) employed a distinct methodology, scrutinizing the prolonged effects of COVID-19 treatment on the male reproductive system. Their investigation, conducted within a timeframe spanning three to eight months post COVID-19 symptom resolution, unveiled that COVID-19 and its therapeutic interventions did not exert enduring impacts on serum androgen levels. Nevertheless, further extensive clinical studies are imperative to corroborate and reinforce our findings.

Guo *et al.* (2021) assessed hormone levels in patients who had tested positive for COVID-19, with an average of 76 days elapsing after the onset of symptoms and 56 days following hospital discharge. In their analysis, testosterone levels, luteinizing hormone (LH), and follicle-stimulating hormone (FSH) exhibited predominantly normal ranges. However, elevated prolactin levels were observed, which could potentially give rise to various physiological conditions, or these heightened levels may have been pre-existing in the patients, as no discernible evidence of recovery was observed in the subsequent sample collection.

Conversely, the findings from the study by Ma *et al.* (2020) present a divergence, wherein the authors reported increased LH levels and a notable decrease in the ratio of testosterone (T) to LH in men who had been infected with SARS-CoV-2. Collectively, these outcomes suggest a plausible impairment of Leydig cells. Acute inflammation, often accompanied by elevated C-reactive protein (CRP) and aberrant cytokine profiles, could potentially influence testicular function and spermatogenesis.

These divergent outcomes might be attributed to variations in the time elapsed between the cessation of COVID-19 symptoms and the collection of the samples. Ma *et al.* (2020) samples were collected from patients actively infected with SARS-CoV-2, whereas the studies examined within this review gathered samples post-recovery from the disease. Consequently, further investigations are warranted, particularly to examine the same individuals at distinct time intervals following SARS-CoV-2 infection (Gul *et al.*, 2021; Guo *et al.*, 2021; Koç & Keseroğlu, 2021).

It is recognized that certain viruses can negatively impact male fertility, albeit the precise mechanisms remain incompletely understood (Lins *et al.*, 2022). An array of microorganisms, spanning bacteria, viruses, and protozoa, may infiltrate the male reproductive tract, leading to seminal quality deterioration. Furthermore, a diverse spectrum of virus families, including mumps, human immunodeficiency virus, human herpes virus, Ebola, and Zika, have been detected in human semen subsequent to infection (Liu *et al.*, 2018).

In the context of SARS-CoV-2 infection, the heightened expression of angiotensin-converting enzyme 2 (ACE2) in Leydig cells, Sertoli cells, and germ cells renders the testes highly susceptible to SARS-CoV-2 infection and resultant damage (Lins *et al.*, 2022). While two studies within the existing literature detected viral RNA in the semen of patients, the prevalence of such instances remained limited. Gacci *et al.* (2021) reported the presence of SARS-CoV-2 in the semen of a solitary patient (2.3%), observed 21 days after a second negative reverse transcription-polymerase chain reaction (RT-PCR) test, while Li *et al.* (2020) identified SARS-CoV-2 in the semen of 4 out of 15 patients (26.7%) in the acute stage of the infection and 2 out of 23 patients (8.7%) who were in the recovery phase.

Nonetheless, the data presented in this review reveals an alternative perspective, as the majority of the studies reported no detectable RNA through RT-PCR in semen, even within samples collected during acute COVID-19 infection (Best *et al.*, 2021; Holtmann *et al.*, 2020; Pan *et al.*, 2020). The paucity of results derived from a broader sampling scope precludes definitive assertions regarding the presence of SARS-CoV-2 in semen and its potential for sexual transmission. As of the present moment, scientific evidence substantiating such transmission remains absent (Silva *et al.*, 2021).

## CONCLUSION

This study has explored potential connections between SARS-CoV-2 infection, diminished seminal quality, and hormonal fluctuations, though it’s crucial to underscore the lack of uniformity in these correlations. Varied research methodologies employed, and the relatively recent emergence of this infection collectively underscore the need for continued investigation.

As a novel health threat, the precise and comprehensive consequences of COVID-19 remain indeterminate. Consequently, the imperative for further research persists, aiming to comprehensively evaluate both the extent and the specific populations potentially impacted by COVID-19. This ongoing inquiry will be instrumental in advancing our understanding of the implications of this global health crisis.

## LIST OF ABBREVIATIONS

Coronavirus 2019: (COVID-19)

Follicle stimulating hormone: (FSH)

Luteinizing Hormone: (LH)

Testosterone: (T)

Angiotensin-Converting Enzyme 2: (ACE2)

Reactive Oxygen Species: (ROS)

Ribonucleic acid: (RNA)
